# Patterns in Genome-Wide Codon Usage Bias in Representative Species of Lycophytes and Ferns

**DOI:** 10.3390/genes15070887

**Published:** 2024-07-05

**Authors:** Piaoran Xu, Lijuan Zhang, Liping Lu, Yanli Zhu, Dandan Gao, Shanshan Liu

**Affiliations:** 1 China-Malaysia National Joint Laboratory, Biomedical Reserch Center, Northwest Minzu University, Lanzhou 730030, China; 18854255615@163.com (P.X.); 290162627@xbmu.edu.cn (L.L.); 294239005@xbmu.edu.cn (Y.Z.); 2 College of Life Science and Engineering, Northwest Minzu University, Lanzhou 730030, China; 289192722@xbmu.edu.cn

**Keywords:** lycophytes, ferns, genetic code, codon usage bias

## Abstract

The latest research shows that ferns and lycophytes have distinct evolutionary lineages. The codon usage patterns of lycophytes and ferns have not yet been documented. To investigate the gene expression profiles across various plant lineages with respect to codon usage, analyze the disparities and determinants of gene evolution in primitive plant species, and identify appropriate exogenous gene expression platforms, the whole-genome sequences of four distinct species were retrieved from the NCBI database. The findings indicated that *Ceratopteris richardii*, *Adiantum capillus-veneris*, and *Selaginella moellendorffii* exhibited an elevated A/U content in their codon base composition and a tendency to end with A/U. Additionally, *S. capillus-veneris* had more C/G in its codons and a tendency to end with C/G. The ENC values derived from both ENC-plot and ENC-ratio analyses deviated significantly from the standard curves, suggesting that the codon usage preferences of these four species were primarily influenced by genetic mutations and natural selection, with natural selection exerting a more prominent influence. This finding was further supported by PR2-Plot, neutrality plot analysis, and COA. A combination of RSCU and ENC values was used as a reference criterion to rank the codons and further identify the optimal codons. The study identified 24 high-frequency codons in *C. richardii*, *A. capillus-veneris*, and *Diphasiastrum complanatum*, with no shared optimal codons among the four species. *Arabidopsis thaliana* and *Ginkgo biloba* exhibited similar codon preferences to the three species, except for *S. moellendorffii*. This research offers a theoretical framework at the genomic codon level for investigating the phylogenetic relationships between lycophytes and ferns, shedding light on gene codon optimization and its implications for genetic engineering in breeding.

## 1. Introduction

Ferns and lycophytes are non-flowering vascular plants, comprising around 13,000 species occupying diverse ecological niches in the temperate and tropical regions of the world [1,2]. Ferns and lycophytes both have ancient plant lineages dating back to the Devonian or earlier and were historically classified as the paraphyletic group ‘pteridophytes’ because they share many similar biological features [1]. With the continuous deepening of phylogenetic research, the major phylogenetic structure of land plants is now becoming clear: ferns and lycophytes have distinct evolutionary lineages, with ferns as the sister group to seed plants, whereas lycophytes represent the sister group to the clade that includes ferns and seed plants.

Although ferns and lycophytes are not as wildly diverse as seed plants, their biology is unique and has a crucial role in our understanding of the evolution, diversification, and origins of land plants. The genetic code contained in the genome determines the differences between species and individuals and is the root of phenotypic generation inheritance and biological evolution. One set of genetic codes weaves together tens of millions of different species, which is a miracle of nature. The four plants examined in this research are notably representative. Presently, *A. capillus-veneris* and *C. richardii* are frequently employed as standard plants in diverse research areas to elucidate the ancestry and biological development of ferns and other phenomena [3,4]. *S. moellendorffii* is also acknowledged as an emerging standard plant for lycophytes [5]. Furthermore, the complete genomes of *D. complanatum* and *S. moellendorffii* at the chromosomal level have been documented. The objective of this research is to examine the codon biases of four specific species, explore the genetic variances between ferns and lycophytes during biological evolution, offer a distinctive theoretical framework for the current classification system of global lycophytes and ferns within the realm of plant whole-genome research, and address challenges related to deciphering the phylogenetic connections among the four selected species based on codon usage patterns, as well as identifying suitable vectors for expressing exogenous gene in the plant genome. Codons play an irreplaceable role in the transmission of genetic information, as a link between amino acids, proteins, and genetic material in living organisms. Degeneracy in codons leads to amino acids being generally encoded by more than one triplet sequence except for methionine (Met) and tryptophan (Trp). And codons that encode the same amino acid are called synonymous codons [6].

Research has shown that the uneven use of synonymous codons is ubiquitous in living organisms [7]. The phenomenon of a species or a gene usually tending to use one or several specific synonymous codons is called codon usage bias (CUB). The generation of codon bias is mainly affected by the interaction of mutation pressure and natural selection. In addition, it is also related to gene length, gene function [8], base composition [9], mRNA secondary structure [10], tRNA abundance [11], and other factors. By analyzing and studying codon usage preference, the codon usage characteristics of species can be described, revealing biological gene evolution, as well as the regulatory mechanisms used during the expression process, which also provide essential references for the expression [12] and prediction [13] of gene functions.

Lycophytes and ferns have essential ecological and economic value [14,15]. In recent years, due to the destruction caused by human excavation and the changes in the geographical environment, the population distribution of lycophytes and ferns has been shrinking, and the number of endangered species in the country has been increasing. A large number of scholars have devoted themselves to identifying the genetic relationships between different species [16,17] and analyzing the phylogenetic relationships [18,19,20] and plastid genome structure variations among the world’s lycophytes [21,22,23] based on the plastid genome sequence. However, there are few studies on interspecific relationships and evolution in lycophytes and ferns at the codon level.

Based on the sequencing results for the genomes of four different genera of lycophytes and ferns, this study compared and analyzed their preferred codon usage patterns, while exploring the influences that affect differences in the preferential use of codons. Our aim was to provide a theoretical basis for the construction and improvement of exogenous genes and expression vectors in the genomes of the four lycophytes and ferns for applications in species conservation, ecological adaptive evolution, codon optimization, and genetic engineering.

## 2. Materials and Methods

### 2.1. Coding Sequence Data

The whole-genome sequences of *A. capillus-veneris*, *C. richardii*, *D. complanatum*, and *S. moellendorffii* were downloaded from the NCBI database (GenBank accession number were GCA_014529385.2, GCA_020310875.1, GCA_029204225.1, and GCA_000143415.2). To reduce the error, their gene coding sequences (CDSs) were screened under the following conditions: the total number of bases in each CDS sequence should be an integer multiple of three; the sequence length should be ≥300 bp; sequence base types should contain only A, U, C, G; each sequence should contain an initiation codon (AUG) and an end codon (UAG, UGA, and UAA); and there should be no termination codon in the middle of the sequence [24,25,26,27]. Eventually, 26,260, 70,423, 67,593, and 27,073 CDS sequences of *A. capillus-veneris*, *C. richardii*, *D. complanatum*, and *S. moellendorffii* were retained, respectively, for subsequent analyses.

### 2.2. Analysis of Codon Composition

The acquired sequences were collated and analyzed using Codon W and Python script, respectively, to obtain the RSCU, T3s, C3s, A3s, G3s, L_sym, L_aa, Gravy, Aromo, CAI, CBI, Fop, and ENC parameters of the relevant sequences. The G/C content of diverse codon positions (denoted as GC1, GC2, and GC3) and the average GC content of the three positions in the genome sequences of four species were, respectively, calculated using the Perl. Statistical analysis of effective codon count (ENC), GC content, codon number (N), and relative synonymous codon usage (RSCU) for each CDS were conducted using Graphpad 10. Among them, RSCU is a statistical index to measure the relative frequency of each synonymous codon [28]. If the RSCU value of a particular codon is equal to 1, this indicates that the codon is used randomly; that is, each synonymous codon is used with the same frequency; RSCU > 1 indicates that the codon is a high-frequency codon, and RSCU < 1 indicates that the codon is a low-frequency codon. Finally, heat mapping, as well as correlation analyses, was carried out using the R language.

### 2.3. ENC-Plot Analysis

ENC was used to evaluate the degree of codon usage bias at the genome-wide level, ranging from 20 to 61. GC3s is the proportion of the G + C content of codon three of the CDS sequence to the total number of bases and is an important index to reveal the preference of nucleotide proportion. The relationship between codon usage bias and base composition was analyzed by plotting GC3s values as horizontal coordinates and ENC values as vertical coordinates. When mutational pressure plays an important role in shaping codon usage patterns, ENC values lie on, or are distributed around, the expected curve. In contrast, when codon usage is affected by factors such as natural selection, ENC values are well below the expected curve [29].

### 2.4. PR2-Plot Analysis

PR2-plot analysis uses G3/(G3 + C3) as the abscissa and A3/(A3 + T3) as the ordinate to draw a scatter plot to analyze the uses and relationship of purines and pyrimidines at 3rd base of the genomic codons. Based on the proportions of A, T, G, and C in the base composition, we can speculate on the magnitude of the effect of base mutations on nucleotide base variation. If the proportions of G and C (or A and T) are similar, then gene codon usage bias is completely affected by mutational pressure; if the proportions of their compositions differ too much, this indicates that codon usage bias is influenced by a combination of natural selection and other factors [30].

### 2.5. Neutrality Plot Analysis

The GC12 value and GC3 value of the genome were calculated using the Perl script. We conducted a neutral plot analysis with GC12 as the ordinate and G3s as the abscissa to analyze the correlation between GC12 and GC3s. When the slope of the regression curve is 0 and there is no significant correlation between GC12 and GC3, this indicates that it is entirely influenced by natural selection; when the slope is 1 and the correlation is significant, this suggests that mutation pressure may be the only driving force. It is used to measure the extent to which natural selection pressure and mutation affect codon usage bias [31].

### 2.6. Correspondence Analysis (CoA)

Correspondence analysis is a multivariate statistical method widely used to explore changes in RSCU and the distribution of genes in multidimensional space [32,33,34,35]. A series of orthogonal axes were generated based on 59 codons (excluding Met, Trp, and termination codons) to reflect trends in codon usage changes, where the percentage of Axis 1 represents the factor that has the greatest impact on changes in codon usage frequency, and the remaining 58 axes represent factors with decreasing influence. CoA can reveal major influences of codon usage patterns in CDS sequences.

### 2.7. RSCU and Optimal Codon Analysis

Referring to the method of Sharp et al. [36], the RSCU was used as an indicator to measure the codon usage bias of four representative species of lycophytes and ferns. The high-frequency codons common to all CDS sequences for each genome were screened, and then the ENC value was used as the screening criterion to rank the codons. The 10% sequences with the highest and lowest ENC values were selected as the high- and low-gene-expression groups, respectively. Then, the RSCU values for 59 codons of the two sets of sequences were calculated, and the ΔRSCU values of codons were calculated to characterize the differences in ENC. Taking ΔRSCU = 0.08 as the critical value, the codons with ΔRSCU ≥ 0.08 and RSCU > 1 in the high-expression group were selected as the high-expression superior codons [37].

### 2.8. Comparison of Codon Usage Preferences between Four Representative Species and Other Plants

The codon usage data of major representative groups of gymnosperms and angiosperms such as *A. thaliana* and *G. biloba* were download from the Codon Usage Database (http://www.kazusa.or.jp/codon/ accessed on 25 June 2023) and compared with the genome codon usage of the four species in this study. When the ratio of the codon usage frequencies of two organisms is ≥2 or ≤0.5, this indicates that the codon usage bias of the two organisms is significantly different [38].

## 3. Results

### 3.1. Codon Composition Analysis

All codons from the genes of four species of lycophytes and ferns were treated with python script, and the specific results are shown (Table 1). The GC content of the first base in the codon was found to be greater than 50% in all four species by analysis, and the GC content of the three codons of most genes is non-uniformly distributed. The GC content distribution trend of *A. capillus-veneris* and *C. richardii* is GC1 > GC3 > GC2; in *D. complanatum*, it is GC1 > GC2 > GC3; and in *S. moellendorffii*, it is GC3 > GC1 > GC2. It can be seen that the codon bases C and G of *A. capillus-veneris*, *C. richardii*, and *D. complanatum* are more likely to appear in the anterior position of each codon. The sequence of the whole genome of *S. moellendorffii* is rich in G/C bases, and the third base of the codon tends to end in C/G. The average GC contents of *A. capillus-veneris*, *C. richardii*, and *D. complanatum* are all less than 50%, indicating that the whole-genome codons of these three species tend to use A/U.

The whole-genome sequences of the four screened plants were analyzed by Codon W [39]. After removing non-synonymous codons and termination codons from the sequences, we found that the T3 and A3 contents were higher than the C3 and G3 contents in *A. capillus-veneris*, *C. richardii*, and *D. complanatum* (Table 2). This shows that among the synonymous codons encoding amino acids, the third base of the codon of *A. capillus-veneris*, *C. richardii*, and *D. complanatum* are mainly dominated by the A/U ending. In *S. moellendorffii*, the G3 and C3 contents were higher than the A3 and T3 contents, indicating that the third base of the codon in the synonymous codon coding for amino acids tended to end with C/G.

### 3.2. ENC Analysis

The distribution of the gene ENC of the four species ranged from 20 to 61, with an average ENC of 50.0 to 53.5 (Table 1). Using ENC = 35 as a criterion for distinguishing the strength of preference, there are 59 *A. capillus-veneris* genes with ENC < 35, accounting for 1.12% of the total. The *C. richardii* gene has 552 entries with ENC < 35, which is 3.92% of the total. The *D. complanatum* gene has 41 entries with ENC < 35, representing 0.30% of the total. The 175 entries with ENC < 35 in the *S. moellendorffii* gene represented 3.23% of the total. In summary, *S. moellendorffii* has the strongest codon usage bias and *D. complanatum* has the weakest codon usage bias compared to the other species. The codon preferences of the four species genes are weak overall, and only a few genes have codon preferences, but there are still differences in codon use preferences among different genes.

### 3.3. Genomic Codon Usage Bias Analysis

The CAI value represents the codon adaptation index and can predict gene expression to a certain extent. The CAI value generally ranges from 0 to 1, and the closer it is to 1, the stronger the codon usage preference. The CAI values of the four species genes ranged from 0.19 to 0.22, indicating that the codon usage bias of coding genes in the four species was generally weak.

The CBI value represents the codon usage bias index, which reflects the proportion of highly expressed codons in a gene. The larger the CBI value in the range from 0 to 1, the stronger the codon usage bias; if the CBI value is less than 0, then the codon usage bias is weaker and is lower than the average frequency of codon usage. Observation of the data shows that the CBI values of *A. capillus-veneris*, *C. richardii*, and *D. complanatum* are less than 0, indicating weak codon usage bias (Table 2). The *S. moellendorffii*, on the other hand, has a CBI value greater than 0, which is a strong codon preference compared to the other three species [40].

The Fop value refers to the frequency of optimal codon usage, representing the ratio of the optimal codon to its synonymous codons. The value range is also from 0 to 1; the larger the value, the stronger the codon usage bias. When the value is 0, it means that the optimal codon is not used, while, when the value is 1, this means that only the optimal codon is used [41]. The Fop values for *A. capillus-veneris*, *C. richardii*, and *D. complanatum* were all around 0.3, with a similar range of values. The Fop value of *S. moellendorffii* was greater than 0.4, again indicating a stronger codon usage bias in *S. moellendorffii* compared to the other species.

### 3.4. Analysis of Factors Influencing Codon Usage Bias

#### 3.4.1. ENC-Plot 

**Figure 1:**
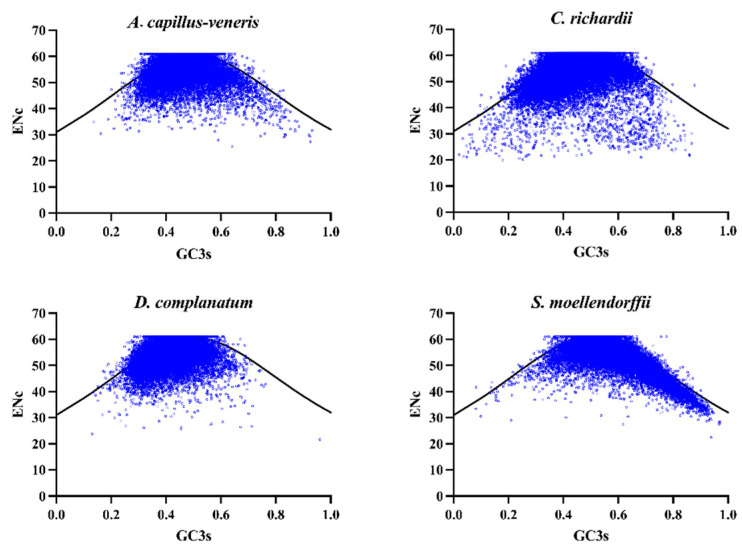
ENC-Plot of four lycophyte and fern genomes. The genes for each species are shown in blue. The GC (ref) line—shown in black—marks the expected location of genes whose codon usage is only determined by the GC content at the third position of a codon (GC3s).

Taking GC3 as the abscissa and ENC as the ordinate, the coding genes of the four species were analyzed through ENC-plot mapping. Most of the genes in the four species were located far below the standard curve, indicating that the ENC values of most of the genes differed from the expected ENC values (Figure 1). Statistical analysis of the ENC ratios of genes showed that the frequency of genes with ENC ratios distributed in the interval from −0.05 to 0.05 ranged from 0.35 to 0.45, indicating that the actual ENC values of these genes were closer to the theoretical ENC values, with less pressure from natural selection and more pressure from mutation. However, there is a greater proportion of gene frequencies with ratios outside the −0.05 to 0.05 interval, suggesting that the actual ENC is more different from the theoretical ENC (Table 3). In other words, it is further away from the standard curve, indicating that these genes are subject to more natural selection pressures. In summary, the codon usage bias of the four species genomes was affected by both mutational and natural selection pressures, but the impact of natural selection was more significant.

#### 3.4.2. PR2-Plot

**Figure 2:**
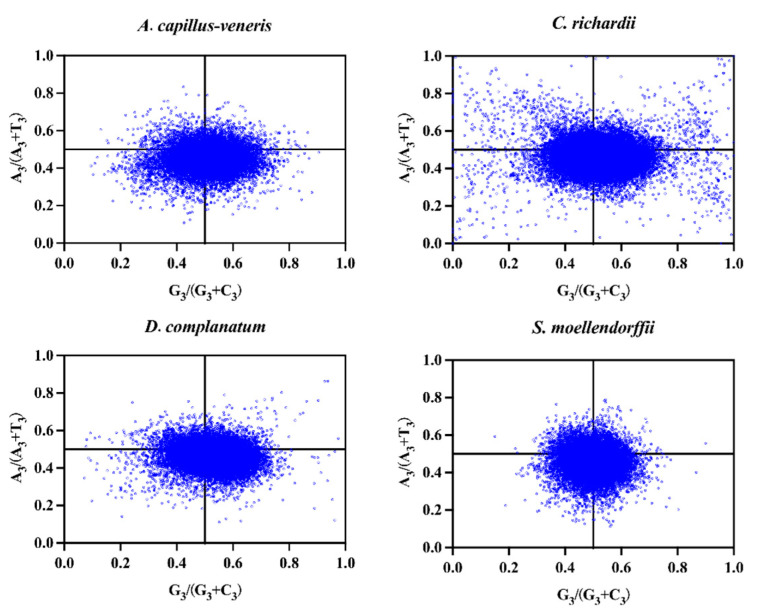
PR2-plot for four lycophyte and fern genomes, plotted according to the GC bias [G3/(G3 + C3)] and AU bias [A3/(A3 + T3)] of CDS at the third-codon position.

Purine (A and G) and pyrimidine (U and C) usage patterns at the third base of codons in genomic sequences were analyzed using parity preference. When mutational pressure alone affects codon usage bias, the randomness of mutation makes the probability of A/U or C/G at the third base of the codon equal, while selection pressure can make the use of A/U or G/C uneven. The coordinate points of the coding genes of the four species are not uniformly distributed in the four areas, with more genes located in the lower right area. Overall, this indicates that base three of the codon is used more frequently in U than in A and more frequently in G than in C (Figure 2). Among four species, the *C. richardii* genome codons are more dispersed and more significantly affected by natural selection. The PR2-plot analysis results show that the codon usage bias in the four species genomes is not only affected by mutations but is also influenced by natural selection and plays an important role in the combination of other factors.

#### 3.4.3. Neutrality Plot

**Figure 3:**
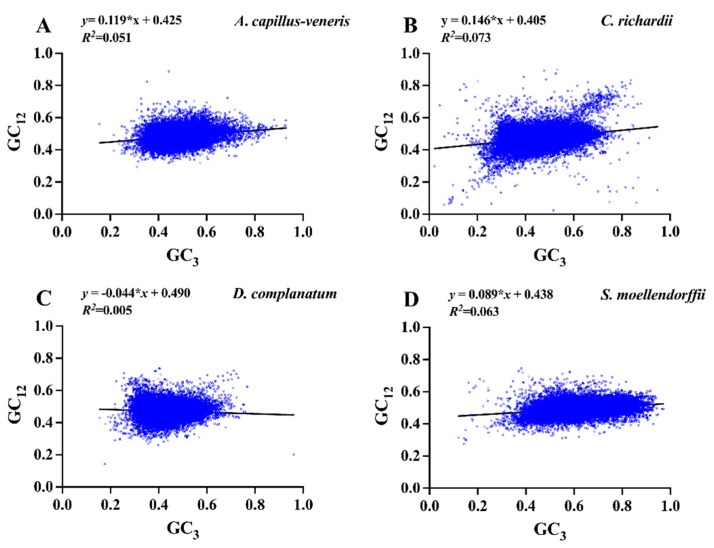
Neutrality plot for four lycophyte and fern genomes. The simulated regression lines are shown in black and represents the actual relationship between GC12 and GC3 values.

Neutral analysis based on GC12 and GC3 can quantitatively evaluate the effects of stress mutations and natural selection. If the slope of the regression curve is close to 1 and the genes are almost equally distributed along the diagonal, it means that the codon usage bias is only affected by mutational pressure; as the slope gradually decreases, even to zero, the effect of natural selection on the codon usage bias will gradually increase. Our results showed that the GC12 values of codons in the four species genomes were distributed between 0.3 and 0.6, and GC3 values were distributed between 0.2 and 0.8. The GC3 value is more often distributed between 0.35 and 0.95 in *S. moellendorffii*, indicating that the third base is used more frequently for G/C than for A/U. The slopes of regression lines for the four species genome ranged from −0.04 to 0.15, with *C. richardii* having a higher slope (0.146) than the other three species and *D. complanatum* having the regression line slope that was closest to zero (−0.044) (Figure 3).

Meanwhile, the correlation between GC12 and GC3 is weak in all four species (r1 = 0.23, r2 = 0.27, r3 = 0.07, r4 = 0.25). Only the GC12 value of *D. complanatum* is negatively correlated with the GC3 value, while the other three show a positive correlation. It can be seen from the above data that mutation pressure only accounts for 4.4–14.6% of the codon usage patterns of the four species, while factors such as natural selection account for 85.4–95.6%, which shows that mutation pressure has little effect on codon usage patterns and that other factors, such as natural selection, play a very important or even dominant role in codon usage patterns.

#### 3.4.4. Correspondence Analysis (CoA)

**Figure 4:**
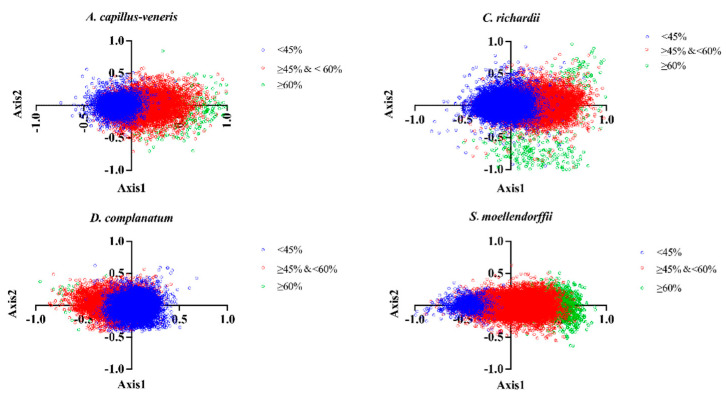
Correspondence analysis of four lycophyte and fern genomes. Figure 4 shows the distribution of genes on the major (Axis 1) and minor axes (Axis 2). GC% < 45% genes are shown in blue, GC% ≥ 45% and <60% genes are shown in red, and GC% ≥ 60% genes are shown in green.

The RSCU distribution of each gene codon in the four species was analyzed in the 58-dimensional vector space to explore the main factors affecting the codon usage variation in these species. The CDS sequences of the four species are distributed on a plane with the first principal factor axis as the abscissa and the second principal factor axis as the ordinate, and the origin represents the average RSCU for all genes relative to axes 1 and 2. Axis 1 accounts for 3.68%, 4.08%, 1.92%, and 6.34% of the total variation in the four species genomes, respectively, while the other axes represent less than 1.5% of the total variation. Exceptionally, the remaining axes of *D. complanatum* account for less than 1.0% of the total variation (Figure 4). This suggests that the codon usage bias characteristics of the four species genes are not influenced by a single factor but are the result of a combination of multiple factors.

Axis 1 occupancy is the most significant effect factor contributing to the variation. In addition, genes were labeled in blue (GC% < 45%), red (GC% ≥ 45% & <60%), and green (GC% ≥ 60%) to explore the effect of the size of the GC content on codon usage preference. No species showed obvious genetic separation in the range of GC content between 45 and 60% and GC content below 45%; *A. capillus-veneris*, *C. richardii*, and *D. complanatum* had a few genes with a GC content greater than or equal to 60%. The difference is that in *D. complanatum*, genes with GC% < 45% are located on the right side of the axis, while genes with GC content between 45% and 60% are located on the left side of the axis; the opposite is true for the other three species. Meanwhile, genes with a GC content greater than or equal to 60% in *C. richardii* showed a relatively dispersed distribution. These phenomena show that the process of codon usage bias formation in the genomes of the four species is complex, and the factors affecting the formation of codon usage bias in different species are not unique.

### 3.5. RSCU and Optimal Codon Analysis

Analysis of the relative usage of synonymous codons in the CDS sequences of four species showed that among the 59 synonymous codons, the high-frequency codons with RSCU > 1 were 28, 28, 29, and 31 in *A. capillus-veneris*, *C. richardii*, *D. complanatum*, and *S. moellendorffii*, respectively (Table 4). *A. capillus-veneris*, *C. richardii*, and *D. complanatum* had 75%, 89%, and 86% of the total number of high-frequency codons ending in A/U; among the total number of high-frequency codons, there were 24, with 87.5% of codons ending in A/U. These four species share nine high-frequency codons. *S. moellendorffii* had 25 high-frequency codons ending in C/G, accounting for 81% of the total number of high-frequency codons. In summary, in all four species, codons in the genomes of *A. capillus-veneris*, *C. richardii*, and *D. complanatum* tended to end in A/U, whereas codons in the genome of *S. moellendorffii* tended to end in C/G.

In the *A. capillus-veneris*, *C. richardii*, *D. complanatum*, and *S. moellendorffii* genomes, the RSCU value ranges are 0.411–1.502, 0.353–1.624, 0.313–1.609, and 0.404–1.56, respectively. The CUU that encodes Leu showed the strongest preferences in both *A. capillus-veneris* and *D. complanatum*, and the ACA that encodes Thr showed the strongest preferences in *C. richardii* and similar preferences in *A. capillus-veneris* and *D. complanatum*. Although the UUG encoding Leu showed the strongest preferences in *S. moellendorffii*, the degree of preference was broadly similar in the other three species (RSCU around 1.2 to 1.3). The GCG encoding Ala was the lowest in codon usage bias in *A. capillus-veneris*, *C. richardii*, and *D. complanatum*, while the GUA encoding Val was the lowest in codon usage bias in the *S. moellendorffii* genome. To summarize, the genomes of *A. capillus-veneris*, *C. richardii*, and *D. complanatum* have a very high similarity in terms of codon usage type and number, while the codon usage pattern in the genome of *S. moellendorffii* is quite different from that of the other three species.

The optimal codon analysis results showed that the high-expression codons with ΔRSCU ≥ 0.08 in the *A. capillus-veneris*, *C. richardii*, *D. complanatum*, and *S. moellendorffii* genomes were 11, 27, 25, and 29, respectively (Table S1). Combined with the high-frequency codons selected by the relative synonymous codon usage, the optimal codons for *A. capillus-veneris* were finally screened out to be five (AAG, UUG, CGC, UCU, and GUG); *C. richardii* and *D. complanatum* have the same 23 optimal codons (GCA, GCU, UGU, GAU, UUU, GGU, CAU, AUU, CUU, AAU, CCA, CCU, CAA, AGA, CGA, CGU, AGU, UCA, UCU, ACA, ACU, GUU, and UAU), all of which end with A/U. *S. moellendorffii* has 25 optimal codons (GCG, UGC, GAC, GAG, UUC, GGC, CAC, AUC, AAG, CUC, CUG, UUG, AAC, CCG, CAG, AGG, CGC, CGG, AGC, UCC, UCG, ACG, GUC, GUG, and UAC), all of which end with C/G. *A. capillus-veneris* and *C. richardii*, *D. complanatum* share one optimal codon for UCU that encodes serine. *A. capillus-veneris* and *S. moellendorffii* share four optimal codons, which are AAG encoding lysine, UUG encoding leucine, CGC encoding arginine, and GUG encoding valine.

### 3.6. Comparison of Codon Usage Patterns between Four Lycophytes and Ferns and Other Species

We compared codon usage patterns in four species and other species including *A. thaliana* (an angiosperm) and *G. biloba* (a gymnosperm) (Table S2). Taking ratio ranges higher than 2.00 or lower than 0.50 as the reference values, we can see the codon usage patterns of *A. capillus-veneris*, *C. richardii*, and *D. complanatum* are extremely similar to those of *A. thaliana* and *G. biloba*; in particular, the ratio of the frequency of occurrence of each codon in *C. richardii* to *A. thaliana* and *G. biloba* is in the range of 0.50 to 2.00. Nevertheless, *S. moellendorffii* presents significant codon preference differences with the other two species and presents as being the least similar to *A. thaliana*.

## 4. Discussion

Codon usage not only reflects the origin, evolution, and mutation patterns of a species or gene but also has an important impact on gene function and protein expression. This study analyzed the codon usage traits of four representative species of lycophytes and ferns and found that *A. capillus-veneris*, *C. richardii*, and *D. complanatum* are highly similar in codon usage patterns. The difference in the total base composition of these three species is small, with all of them being higher in A/U and lower in G/C; all of the G/C are more often distributed on the first base of the codon, and they all tend to end in A/U. Similar results have been found in other species, such as *Aconitum* [42], *Sarcozygium xanthoxylon Bunge* [43], *Chlorella sorokiniana* [44], and *Cyanobacteria* [45]. On the other hand, *S. moellendorffii* differs from the other three in its codon usage pattern, in that the total base composition has a higher G/C content and tends to end in G/C. The results of the study are in agreement with those of Zhang et al. [21]. This finding provides additional evidence of the distinctive characteristics of *S. moellendorffii* in the evolutionary development of lycophytes, particularly in terms of codon content and composition. As a newly recognized model organism within the lycophyte group, *S. moellendorffii* occupies a significant evolutionary position.

A comparative analysis of the RSCU values revealed that there are 24 preference codons shared by *A. capillus-veneris*, *C. richardii*, and *D. complanatum*, of which 87.5% ended in A/U. However, when added to the combined analysis of *S. moellendorffii* data, there were only nine preference codons shared by the four, and *S. moellendorffii* preference codons ending in C/G accounted for 81% of the total number of preference codons. It is shown that there is a high degree of consistency in GC content and codon usage among *A. capillus-veneris*, *C. richardii*, and *D. complanatum*, with *S. moellendorffii* differing significantly from the other three. Research shows that the GC content of monocots is significantly higher than that of dicots, so dicot nuclear genes tend to end in A or U, while monocots tend to end in C or G [46]. When the average GC and GC3 content in some medicinal plants is about 50%, the genome does not show obvious codon usage bias, indicating that base composition plays an important role in shaping codon preference.

In addition, PR2-Plot, ENC-Plot, neutrality plot analyses, and correspondence analysis were performed on the codons in the genomes of the four species to better understand the factors affecting codon usage bias. The results showed that the four species of lycophytes and ferns were more influenced by natural selection. According to the PR2-plot mapping analysis, the third-position bases of the four species were found to have certain preferences in codon usage, with the main preferences being as follows: U > A, G > C. However, some genes do not fit neatly into being affected by selection alone, suggesting that codon usage bias is also affected by mutational pressure, as well as other factors, and that the strength of mutational pressure also affects the strength of the preference.

In this study, high-frequency codons and high-expression codons were used as criteria for screening the optimal codons, and, finally, five optimal codons for *A. capillus-veneris*, 23 optimal codons for *C. richardii* and *D. complanatum*, and 25 optimal codons for *S. moellendorffii* were screened out, and the four species do not share optimal codons with each other. The lower number of optimal codons in *A. capillus-veneris* may be because most of the high-frequency codons end in A/U, whereas most of the high-expression codons end in C/G, or it may be due to mutational pressure. The codon usage bias of *A. capillus-veneris*, *C. richardii*, and *D. complanatum* are extremely similar, so we inferred that these three species may have high similarity in their evolutionary and ecological evolutionary patterns, which may also be related to the strong genomic conservation among related species [47]. In turn, the specific genomic codon composition of *S. moellendorffii* provides clues to its differences from other species in terms of phylogeny and ecological adaptability evolution.

We compared the codon usage patterns of these four species with those of *A. thaliana* and *G. biloba*. With the exception of *S. moellendorffii*, the codon usage of the other three species differed only slightly from those of *A. thaliana* or *G. biloba*. This is in accordance with Pryer et al. [48], who first clarified from a molecular perspective that not all extant ferns are a monophyletic group, with the *lycopodophytes* (including the Lycopinaceae and the Selagopinellaceae) being the earliest evolved groups and sister groups to the other vascular plants.

Research on the transformation of lycophytes and ferns from vascular plants requires the use of more typical plants, and further optimization of these plants is needed. Through this study, we screened recipient plants with greater transformation efficiency for the genetic heterologous transformation of three further plants besides *S. moellendorffii. A. capillus-veneris* is minimally divergent from the angiosperm *A. thaliana* and may be the best recipient plant for verifying the function of its genes. The codon usage pattern of *C. richardii* was almost identical to that of *A. thaliana* and *G. biloba*, both of which represent the best recipient plants. In contrast, the codon preference differences in *S. moellendorffii* were significantly different from those in *A. thaliana* and *G. biloba*. We speculate that *S. moellendorffii*, represented by *Selaginllaceae*, may be more distantly related to gymnosperms and angiosperms. To improve the transformation efficiency of *S. moellendorffii*, as well as to cultivate excellent germplasm resources, further research and exploration into *S. moellendorffii* are needed.

These four representative species of lycophytes and ferns have important ecological and economic value. In recent years, due to human-made mining damage and changes in the geographical environment, the distribution range of plant populations has been decreasing, and the number of nationally endangered species has been increasing. A preliminary assessment of the endangerment level of Chinese lycophytes and ferns according to IUCN grades and standards revealed that *Ceratopteris* and *Selaginella* are vulnerable species in China. Using the codons of representative plants for lycophytes and ferns as research objects, we identified suitable heterologous species for the genetic modification of these species, which is highly important for codon optimization. At the same time, a preliminary exploration of the genetic information of the four species was conducted to provide theoretical support for the later development and utilization of the four species of lycophytes and ferns and to achieve the large-scale propagation of valuable and endangered plants. 

It is noteworthy that there are notable distinctions in codon base composition and base usage patterns between *S. moellendorffii* and *D. complanatum*, two contemporary lycophyte species. The phenomenon known as whole-genome duplication (WGD) or polyploidization has been recognized as a significant factor contributing to the variability in genome size and chromosome number [49]. Homosporous lycophytes (Lycopodiaceae) and heterosporous lycophytes (*Selaginella* and *Isoetes*) exhibit distinct reproductive strategies. Both Lycopodiaceae and Selaginellaceae have undergone separate instances of whole-genome duplication (WGD) throughout their evolutionary history [50]. It is hypothesized that the distinct evolutionary processes of *D. complanatum* and *S. moellendorffii* may account for the significantly larger genome size and chromosome number observed in *D. complanatum*. This divergence likely contributes to the development of distinct codon usage patterns in each species, thereby offering novel avenues for investigating the distinctive genome evolutionary trajectory of ancient lycophytes.

## 5. Conclusions

The research findings indicate that the base composition and utilization of synonymous codons play a significant role in the ongoing biological evolution of lycophytes and ferns. Variations in codon preference profiles between *S. moellendorffii* and the other three species appear to have been partly shaped by mutational pressure and natural selection. Even if natural selection prevails, the intensity of mutational pressure influences the degree of preference, as well. To validate the functionality of diverse species genomes, selecting *A. capillus-veneris*, *C. richardii*, and *D. complanatum* as host plants would be a favorable decision. While codon usage bias is not a compulsory metric for conducting phylogenetic structural studies in terrestrial plants, our research provides insights into ferns and lycophytes within the framework of the evolutionary progression of archaeal plant lineages from a unique standpoint.

## Figures and Tables

**Table 1:** Genomic GC contents and ENC values. The abbreviations of the labels in the first row are explained in the footer of the table.

	GC1	GC2	GC3	GCall	ENC
*A. capillus-veneris*	53.615	42.522	46.740	47.626	52.778
*C. richardii*	51.832	41.442	41.977	45.084	52.283
*D. complanatum*	52.706	41.724	40.989	45.140	53.410
*S. moellendorffii*	55.834	42.535	60.650	53.006	50.498

Abbreviations: GC1, GC content at the first position of a codon; GC2, GC content at the second position of a codon; GC3, GC content at the third position of a codon; GCall, GC content of all codons in the genome; ENC, the effective number of codons.

**Table 2:** Codon index for four representative species of lycophytes and ferns. The collation of data for 13 codon indexes of four lycophytes and ferns enables us to predict genetic differences between the genomes of different species. In the footer of the table, the abbreviations for the labels in the first row and column are explained.

Codon Index	AC	CR	DC	SM
T3s	0.372	0.401	0.406	0.269
C3s	0.270	0.234	0.227	0.371
A3s	0.308	0.344	0.348	0.231
G3s	0.295	0.274	0.269	0.375
CAI	0.200	0.198	0.199	0.216
CBI	−0.064	−0.078	−0.068	0.038
Fop	0.380	0.373	0.379	0.438
GC3s	0.446	0.397	0.388	0.592
GC	0.477	0.452	0.452	0.531
L_sym	413.202	462.340	526.286	414.89
L_aa	429.631	480.550	545.783	431.26
Gravy	−0.253	−0.266	−0.269	−0.226
Aromo	0.079	0.082	0.080	0.082

Abbreviations: AC, A. capillus-veneris; CR, C. richardii; DC, D. complanatum; SM, *S. moellendorffii*; T3s/C3s/A3s/G3s, frequency of T/C/A/G at the third base of codons; CAI, codon adaptation index; CBI, codon bias index; Fop, frequency of optimal codons; GC3s, G + C content at the third positions of the synonymous codon; GC, GC content of genes; L_sym, number of synonymous codons; L_aa, total number of amino acids; Gravy, grand average of hydropathicity; Aromo, aromatic protein influence codon usage bias parameters.

**Table 3:** The distribution of ENC ratios. The distribution of ENC ratios reflects the extent of deviation between the actual ENC value and the theoretical ENC value of a gene, which can be used to determine the factors influencing codon preference.

Class Limit	Class Value	Frequency				Frequency Rate			
		AC	CR	DC	SM	AC	CR	DC	SM
−0.25~−0.15	−0.2	2	12	15	4	0.00008	0.00017	0.00022	0.00015
−0.15~−0.05	−0.1	254	847	780	180	0.00967	0.01207	0.01154	0.00665
−0.05~0.05	0	9499	27,130	27,822	11,517	0.36176	0.38664	0.41163	0.42541
0.05~0.15	0.1	13,932	37,721	35,779	13,681	0.53058	0.53757	0.52935	0.50534
0.15~0.25	0.2	2043	3414	2746	1513	0.07780	0.04865	0.04063	0.05589
0.25~0.35	0.3	461	496	379	157	0.01756	0.00707	0.00561	0.00580
0.35~0.45	0.4	60	309	51	17	0.00229	0.00440	0.00075	0.00063
0.45~0.55	0.5	7	240	18	4	0.00027	0.00342	0.00027	0.00015
Total		26,258	70,169	67,590	27,073	1	1	1	1

**Table 4:** The RSCU values for the CDS sequence. Codon frequencies in the genes of the four species were counted to determine a set of the most frequently used codons. Number, sum of codon frequencies; RSCU, relative synonymous codon usage. The bold areas indicate RSCU > 1 (high-frequency codon).

Amino Acid	Codon	Number				RSCU			
		AC	CR	DC	SM	AC	CR	DC	SM
Ala	GCA	328,432	989,237	1,115,071	214,300	**1.466**	**1.618**	**1.578**	0.871
	GCC	173,863	376,265	438,127	244,188	0.776	0.615	0.62	0.993
	GCG	91,978	216,035	220,899	248,354	0.411	0.353	0.313	**1.01**
	GCU	301,610	864,565	1,051,993	277,044	**1.347**	**1.414**	**1.489**	**1.126**
Cys	UGC	119,373	318,043	311,419	145,304	**1.022**	0.89	0.922	**1.325**
	UGU	114,153	396,826	364,193	73,974	0.978	**1.11**	**1.078**	0.675
Asp	GAC	200,175	514,792	569,822	311,951	0.691	0.573	0.599	**1.012**
	GAU	378,798	1,281,555	1,331,887	304,291	**1.309**	**1.427**	**1.401**	0.988
Glu	GAA	328,776	1,157,275	1,341,654	272,737	0.911	**1.043**	**1.089**	0.721
	GAG	393,172	1,061,377	1,123,251	483,614	**1.089**	0.957	0.911	**1.279**
Phe	UUC	173,387	536,715	507,514	268,206	0.794	0.814	0.716	**1.129**
	UUU	263,340	782,464	910,388	206,909	**1.206**	**1.186**	**1.284**	0.871
Gly	GGA	213,205	714,541	837,986	250,272	**1.135**	**1.317**	**1.386**	**1.256**
	GGC	182,091	422,746	489,520	242,831	0.97	0.779	0.81	**1.219**
	GGG	155,260	404,290	427,736	157,826	0.827	0.745	0.708	0.792
	GGU	200,691	629,334	662,767	145,882	**1.069**	**1.16**	**1.096**	0.732
His	CAC	108,927	272,492	306,282	165,967	0.712	0.594	0.648	**1.189**
	CAU	197,221	645,662	639,124	113,122	**1.288**	**1.406**	**1.352**	0.811
Ile	AUA	132,939	495,964	476,885	89,876	0.763	0.854	0.788	0.493
	AUC	155,759	472,970	492,669	282,736	0.894	0.815	0.814	**1.55**
	AUU	233,749	772,433	845,577	174,532	**1.342**	**1.331**	**1.398**	0.957
Lys	AAA	264,644	906,517	1,046,153	208,904	0.853	0.954	0.998	0.663
	AAG	356,189	994,699	1,049,801	421,403	**1.147**	**1.046**	**1.002**	**1.337**
Leu	CUA	114,756	342,395	398,912	92,206	0.616	0.644	0.688	0.409
	CUC	168,150	414,007	386,053	331,224	0.903	0.778	0.666	**1.47**
	CUG	182,510	514,216	600,222	277,257	0.98	0.967	**1.036**	**1.231**
	CUU	279,707	857,324	932,495	200,373	**1.502**	**1.612**	**1.609**	0.889
	UUA	123,649	451,252	468,975	60,664	0.629	0.745	0.698	0.44
	UUG	269,356	759,794	874,982	215,282	**1.371**	**1.255**	**1.302**	**1.56**
Met	AUG	282,681	866,305	860,493	276,469	1	1	1	1
Asn	AAC	163,371	483,812	520,991	238,044	0.772	0.696	0.707	**1.195**
	AAU	259,882	906,830	953,288	160,282	**1.228**	**1.304**	**1.293**	0.805
Pro	CCA	184,259	582,251	663,628	178,328	**1.322**	**1.494**	**1.505**	**1.253**
	CCC	109,951	234,875	252,967	115,033	0.789	0.603	0.574	0.808
	CCG	61,060	153,096	151,889	144,537	0.438	0.393	0.344	**1.016**
	CCU	202,036	588,760	695,197	131,240	**1.45**	**1.511**	**1.577**	0.922
Gln	CAA	247,609	716,536	854,813	184,836	**1.049**	**1.027**	**1.043**	0.822
	CAG	224,626	678,388	783,813	264,729	0.951	0.973	0.957	**1.178**
Arg	AGA	163,721	556,336	601,738	137,503	**1.033**	**1.098**	**1.152**	0.924
	AGG	153,253	456,708	443,328	160,121	0.967	0.902	0.848	**1.076**
	CGA	82,637	238,749	295,095	107,147	**1.102**	**1.157**	**1.236**	**1.062**
	CGC	79,522	167,607	190,657	116,118	**1.06**	0.813	0.798	**1.151**
	CGG	65,558	173,829	182,712	111,949	0.874	0.843	0.765	**1.11**
	CGU	72,345	244,908	286,909	68,380	0.964	**1.187**	**1.201**	0.678
Ser	AGC	179,597	440,360	505,404	236,996	**1.054**	0.901	0.924	**1.39**
	AGU	161,155	537,111	588,387	104,037	0.946	**1.099**	**1.076**	0.61
	UCA	205,493	738,956	786,199	102,542	**1.252**	**1.407**	**1.409**	0.671
	UCC	136,411	378,592	385,704	178,370	0.831	0.721	0.691	**1.168**
	UCG	78,268	211,624	222,177	185,204	0.477	0.403	0.398	**1.213**
	UCU	236,121	772,144	837,411	144,767	**1.439**	**1.47**	**1.501**	0.948
Thr	ACA	205,060	670,959	703,525	127,562	**1.495**	**1.624**	**1.551**	0.914
	ACC	115,165	265,042	305,155	131,863	0.839	0.642	0.673	0.944
	ACG	66,861	184,439	189,383	154,925	0.487	0.446	0.418	**1.11**
	ACU	161,738	532,033	615,793	144,121	**1.179**	**1.288**	**1.358**	**1.032**
Val	GUA	128,256	433,311	448,957	82,007	0.692	0.802	0.751	0.404
	GUC	137,417	379,589	378,766	211,259	0.742	0.702	0.633	**1.041**
	GUG	250,181	619,010	715,051	312,706	**1.35**	**1.145**	**1.196**	**1.54**
	GUU	225,220	729,989	848,969	206,157	**1.216**	**1.351**	**1.42**	**1.015**
Trp	UGG	148,751	416,101	457,356	168,797	1	1	1	1
Tyr	UAC	119,310	326,033	350,895	192,562	0.828	0.711	0.744	**1.247**
	UAU	168,731	591,712	592,105	116,394	**1.172**	**1.289**	**1.256**	0.753

## Data Availability

The taxonomic information and genome data that support the findings of this study are openly available in [NCBI] at https://www.ncbi.nlm.nih.gov/ accessed on 25 June 2023. All data generated or analyzed during this study are included in the article and its additional files.

## Supplementary Materials

The following supporting information can be downloaded from https://www.mdpi.com/article/10.3390/genes15070887/s1, Table S1: Relative synonymous usage of high-/low-expression sample group in four lycophyte and fern genomes; Table S2: Comparison of codon preference between four representative species and *A. thaliana* and *G. biloba*.
